# On Modern Progress in Ophthalmic Medicine and Surgery

**Published:** 1896-02-22

**Authors:** Robert Brudenell Carter

**Affiliations:** Consulting Ophthalmic Surgeon to St. George's Hospital.


### On Modern Progress in Ophthalmic Medicine and Surgery.

By Robert Brudenell Carter, F.R.C.S., Consulting Ophthalmic Surgeon to St. George's Hospital.

### LATENT SQUINT (HETEROPHORIA).

In addition to the declared forms of squint, much attention has of late years been given, especially in the United States, to a condition there called heterophoria, but which, having a very strong objection to verbal monstrosities, I prefer to describe as "latent squint." In order thoroughly to understand what this means it is necessary to remember that a person with two good eyes would see the object of vision double unless the two eyes were directed to the same point, whether this were near or distant. With the majority of people the required condition of single vision is easily and naturally fulfilled, the eyes turning together with perfect correctness, and both being naturally directed to the same object. In declared squint, on the other hand, the two eyes are habitually directed to different points, but the squinting eye deviates so much from the direction of its fellow that the image which it receives falls upon a peripheral part of the retina, possessing only a limited degree of sensibility, and is easily neglected by the consciousness; so that double vision, although produced, is unnoticed by the subject, and is no longer irksome. Between these conditions we find one in which the two eyes can be directed to the same point, and habitually are so directed in order to avoid double vision, but in which their tendency is slightly to deviate. Such eyes, instead of having the normal tendency to combination, have a tendency to go astray from one another; and the vicious tendency is only controlled by a special effort of the muscles, which must be made continuously, and which may become exceedingly fatiguing.

The first recognition of latent squint was by the late Albrecht V. Graefe, who described one of its common forms under the name of insufficiency of the internal recti muscles. He found cases in which work with the eyes upon near objects was difficult or impossible, and in which the difficulty could neither be traced to any error of refraction nor to any weakness of accommodation, but only to the effort of maintaining convergence to the position of the book, paper, or other object which had to be steadfastly regarded. The tendency of the eyes was to become divergent, relatively to the object of near vision, although no divergence was apparent on casual inspection. If divergence had been permitted to occur all work would have been rendered impossible by the double vision which must have followed; and hence the eyes were forcibly held right as long as the power so to hold them was retained. The effort, in some persons, occasioned fatigue, headache, and general incapacity, so that "muscular asthenopia" became a generally recognised form of ocular distress. It has since been ascertained that "insufficiency of the interni" does not by any means stand alone, and that the tendency to deviation may be in any direction, upwards, downwards, inwards, or outwards.

When a glass prism is held between the eye and an object of vision, the position of the latter appears to be changed by being moved in a direction away from the base of the prism and towards its angle, and to an extent corresponding with the angular measurement of the prism employed. In consequence of this property of the prism, it has been made available as a means of discovering the resting position of the eyes and the strength of the several ocular muscles.

If the two eyes be directed towards a point of light, preferably at from ten to twenty feet away, and if a prism with its base downwards be held before one of them, the point as seen by that eye will appear to be displaced upwards, while to the other it will remain in its natural position. Double vision will be produced, and two points of light will be seen, one at a higher level than the other. If the prism be not too strong, the patient, under the influence of his knowledge that there is only one point, will try to make what he sees harmonise with what he knows, and will exert the muscles of his eyes in such a manner as to restore single vision. In the case supposed, the eye behind the prism would deviate upwards for this purpose. If the prism were held with its base upwards, the deviation to restore single vision would be downwards. In order to test the strength of the lateral muscles, the prism is held with its base outwards for the interni, and with its base inwards for the externi; but, in both the latter cases, the power of deviation is usually so great that it is necessary to place a prism before each eye in order to attain the required degree. Speaking generally, and subject to certain exceptions, the group of healthy eye muscles should be able to restore single vision notwithstanding prisms of about forty degrees with their bases outwards, and of about seven or eight degrees with their bases inwards. The power of upward or downward rotation can seldom overcome a prism of more than three degrees.

In order to ascertain the tendencies of the eye muscles, in respect of position, it is necessary to employ objects of vision which the patient can accept as correct in the aspects which they present to him. For example, it will often fulfil the required conditions if the patient is furnished with a prism of six degrees, base downwards, before one eye, and with a red glass before the other, in a trial frame. If a point of light is then uncovered, he will see a red point of light with one eye, and a white point of light with the other, the white point appearing to be at a higher level than the red one. He will make no effort to combine these dissimilar objects, so that his eyes will assume their natural position in relation to each other. If this position be correct, the white point of light will appear to be directly above the red one. If the tendency of the eyes be towards convergence, the white point will not be directly above the red one, but will deviate towards the side of the eye to which it belongs. If the tendency be towards divergence, the deviation will be in the opposite direction. Let it be supposed that the prism is before the left eye, and the red glass before the right. A tendency towards convergence will exhibit a red point below, and a white one above it and to the left. A tendency to divergence will reverse the lateral positions, and will send the white point to the right. The amount of the tendency to deviation may be roughly estimated by the apparent distance (laterally) between the two points; or may be measured with tolerable exactness by finding the strength of prism, with its base inwards or outwards, as the case may be, which will restore the two images of the point of light to a correctly vertical position.

